# The Fundamental Code Unit of the Brain: Towards a New Model for Cognitive Geometry

**DOI:** 10.1007/s12559-017-9538-5

**Published:** 2018-01-20

**Authors:** Newton Howard, Amir Hussain

**Affiliations:** 1 The Univeristy of Oxford, Oxford, UK; 2 University of Stirling, Scotland, UK

**Keywords:** Fundamental Code Unit (FCU), Cognitive Geometry, Long-term memory (LM), Long term, Potentiation/depression (LTP/LTD)

## Abstract

This paper discusses the problems arising from the multidisciplinary nature of cognitive research and the need to conceptually unify insights from multiple fields into the phenomena that drive cognition. Specifically, the Fundamental Code Unit (FCU) is proposed as a means to better quantify the intelligent thought process at multiple levels of analysis. From the linguistic and behavioral output, FCU produces to the chemical and physical processes within the brain that drive it. The proposed method efficiently model the most complex decision-making process performed by the brain.

## Introduction

As humans living in a complex world, our success and survival depends on our ability to simplify and understand what we observe in our environment, a process of formulating and reformulating received information into cognitive models and systems. Cognitive agents organize objects, concepts, and ourselves into schemes consisting of fundamental units, which then constitute an overarching structure. Once created, cognitive agents continue to develop and refer to these models with every new experience and observation; in fact, these models could be said to form the basis of all our subsequent cognitive processes. For researchers and theorists working to understand the human brain, the significance of these behaviors supports the effort to identify a “fundamental unit” of thought. By defining and then organizing these units into the larger processes that form human consciousness, we might advance new ways of thinking about cognition and awareness.

Contemporary research (e.g., [1]) has gravitated towards the quantum and electromagnetic explanations of consciousness due to the fact that so little is known about this phenomenon (for a good overview of state-of-the-art, see, Hussain and Howard et al. 2016 [2]). However, because cognition itself is not simply a single natural process but a group of processes that we categorize as composing conscious thought, any attempt to model these processes must take multiple interdependent levels of analysis into account. This approach has led to a fundamental rift between several disciplines that each contributes directly to our understanding of the brain, such as philosophical, psychological, and neuroscientific perspectives [3].

A comprehensive understanding of cognition presupposes more than simply a grasp of the physical and chemical processes at work. The highest, or philosophical, level of analysis serves as an ideal starting point because in order to model cognition, there needs to exist some consensus as to what it is, or at least some criteria that our model must satisfy. Philosophical models such as the duality of mind and brain must frame the discourse on cognition, because intelligent thought does not occur in a vacuum; it needs to be defined in both relative and absolute terms. In addition, conflating the processes that comprise intelligent thought with the perception of those processes by other intelligent thinkers would not lead to an applicable model. In order to usefully quantify the physical processes comprising cognition, we propose a system to analyze the different mediums of brain function in a mathematically uniform manner. This system manifests itself at several levels and ways relating to brain physiology, including neuronal activity, molecular chirality, and frequency oscillations. We argue that to best understand the unitary system, it is imperative to comprehend its expression through each of these mediums instead of focusing on any single level.

The brain contains approximately 100 billion neurons, each of which has roughly the processing capability of a small computer. A considerable fraction of the 100 billion neurons are active simultaneously and do much of their information processing through interactions with one another [4]. Frequency oscillations in neuronal and electronic-related releases are the underlying causes of most brain disorders [5]. As such, it is crucial to understand the nature of such frequencies, their causes, their ranges, and the relation of each range to each disorder. In addition, this understanding will reinforce the psychological analysis as well [6].

The purpose of the current paper is twofold. First, we seek to present the FCU theory in terms that are more accessible to practitioners within a broad range of current scientific endeavor, both basic and applied: neuroscience in particular, but also psychology, sociology, and electrical engineering (e.g., circuit design theory and practice). The second objective is to present, within the framework of this new paradigm, a view of several lines of information emerging from molecular, cellular, and behavioral neuroscience that are both supportive of this new paradigm and—most importantly—suggest how the FCU hypothesis can be tested, the goals being to elucidate the neural correlates of the FCU, and apply this information to new therapeutic strategies as well productively addressing the broader quasi-philosophical questions cited above.

To this end, this paper discusses a novel multi-disciplinary quantum physics approach to developing a theory of concepts that solves the combination problem, i.e., to deliver a description of the combination of concepts. At this level, we can develop a better understanding of the quantum activity needed to affect human behavior in a meaningful way (for an overview of quantum computing and its problem-solving capabilities, see [7]). Specifically, the authors focus on two chemical and physical mechanisms within the brain that have a direct impact on cognitive function. The first is the role of transduction-associated channels in the hippocampal region of the brain that regulate visual stimulus processing. Since these channels rely on similar mechanisms to the neural correlates of language but are much better mapped and known, it is possible to apply what is known about them to the link between language and cognition. Second is the prominence of astrocytes containing key components of an amplification and transduction cascade (a CGMP-triggered transduction channel). These astrocytes are much larger in humans than in primates, suggesting an important neuro-cognitive link [8]. Based on what is known about the physiology of astrocytes, we argue that they may play a key role in signal amplification and transduction, wherever they are found in the brain.

Finally, neuroplasticity-based changes that produce memories, such as long-term synaptic depression (LTP) and long-term synaptic facilitation (LTD), are examined in order to determine FCU-based cognitive variance over long periods of time.

## Analytical Goals

An analytically rigorous, comprehensive approach to modeling human cognition has myriad applications; in addition to better understanding processes such as language acquisition and developmental neurobiology, it can aid in predictive behavioral analysis and organizational dynamics. We must begin by answering two related questions. First, how much neuronal activity constitutes a coherent thought? Understanding patterns of neuronal activity is a prerequisite to determining which aspects of those patterns correspond to the phenomenon of the individual thought, aspects that we hold to constitute the FCU. Second, do some types of neuronal activity compose cognition while others do not? Since the number of neural networks in which any given neuron can exist is limited only by the connections it forms with its neighbors, it is possible that some of its activity contributes to conscious brain function, while some do not. Discerning the type of neuronal activity that contributes to cognition at a single-cellular level will similarly contribute to the search for a fundamental unit that can be used to classify thought.

In addition to examining the composition of cognition at philosophical, psychological, and neurochemical levels through argument, this paper utilizes a series of case studies, or applications of the human brain, to demonstrate the mapping of the physical processes we observe at the chemical level to cognitive changes that are exhibited through behavior, such as the development of language skills and the notion of linguistic semantic primitives [9]. Since these concepts are the building blocks of coherent *communicated* thought, the interface between them and a more basic, fundamental cognitive code can shed important light on human thought processes. Ultimately, the goal of this paper can be applied to three broad conceptual categories:

- FCU as the unit of cognition: to devise a coherent mapping of the FCU to thought components, linguistic constructs and structures, as well as behavior
- Mind/body dualism a defining component of the conceptual framework of cognition: to develop a mathematical framework with explanatory and predictive value, even though it is not possible to directly observe or objectify the concepts explained therein
- To delineate precisely what can be measured within the brain and how—together with external observations of language, behavior—the nature of the FCU can be precisely quantified

## Current Approaches: State-of-the-art

### The Molecular Basis for Cognition

Amino acid molecules are constituted by an amine group, a carboxylic acid group, and a side-chain that varies between different amino acids. The key elements of an amino acid are carbon, hydrogen, oxygen, and nitrogen. The amino acid phenylalanine is of particular importance to the construction of neurotransmitter precursors. There are several important neurotransmitters that merit focus in our discussion of the chemical components of cognition. Some of these major neurotransmitter classes include:

- Amino acids (glutamate, aspartate, D-serine, γ-aminobutyric acid (GABA), glycine)
- Monoamines and other biogenic amines (dopamine (DA), norepinephrine (noradrenaline; NE, NA), epinephrine (adrenaline), histamine, serotonin (SE, 5-HT))
- Others (acetylcholine (ACh), adenosine, anandamide, nitric oxide)

Evidence based on analysis of short-term memory (SM) processes, or “working memory,” shows that persistent firing of the corresponding neuronal networks is required for the encoding of information [10]. If we assume that working memory is always active during conscious moments, this suggests that at the level of the single neuron, state changes are constantly occurring, not unlike memory cells in the random access memory of a personal computer. However, it is unclear whether these state changes necessarily correspond to numerically based distinctions, or whether more subtle state differences are responsible for the communication of information.

### Metabolic Involvement in Conscious Thought

The link between long-term memory (LM) and cellular/synaptic processes such as long-term potentiation/depression (LTP/LTD) [11] requires some sort of structural changes/protein synthesis:

1. *Changing neurotransmitter receptor expression* [12]*,*
2. *Increasing synapse size* [13]*, and*
3. *Changing synapse anchoring* [14]*.*

ADP/ATP (adenosine diphosphate/adenosite triphosphate) represents the major energy source in neurons and glial cells, and is therefore required for long-term memory preservation [15]. Apart from ATP/ADP fuelling persistent activity by driving ATP/ADP-dependent ionic pumps and the maintenance of synaptic receptors, the study of ATP/ADP has shown it to be directly linked to the emergence of persistent activity through its modulation of ATP-modulated potassium channels ([16]. ADP/ATP energy per activation for each unit of actions and columns (assuming C is a fundamental module of the perceptual system) meets the activation requirement as the force for persistent activity.

Recent near-infrared studies have shown that an increase in ATP availability leads to cognitive enhancement [17]. Burnstock discovered this purinergic signaling phenomenon [18], showing the involvement of ATP/ADP-mediated signaling through neuronal and glial receptors in nearly every aspect of brain function. Since his discovery of purinergic signaling, there have emerged numerous studies showing the involvement of ATP/ADP-mediated signaling through neuronal and glial receptors in many aspects of brain function.

### Neurological Signaling

Evolution has provided humans with a multilayer central nervous system; that is, a given neuron can be involved in the transmission of vast numbers of simultaneous signals. By this process, one neuron releases a neurotransmitter into a small space (the synapse) that is adjacent to another neuron. Neurotransmitters must then be cleared from the synapse efficiently so that it can be ready to function again as soon as possible.

In neurons, information is transmitted as “spikes”; it is unclear how exactly these spikes encode information, and there is a continuing debate in the field about whether the timing of these spikes is an important informational component. What comprises these spikes is the activity of voltage-gated ion channels, which are themselves *stochastic mechanisms*, or nonlinear systems [19] that have some significant degree of signal noise.

Since these spikes, or instances of stochastic resonance, are the basis for information transmission in the brain, it follows that an attempt to model cognition from the lowest level up should begin at the signal-unit level. While it is easily discerned that thoughts are necessarily composed of these spikes, and in a literal sense they form the basic units of thought, this perspective offers little new insight—there needs to be some useful, repeated composition of these spikes that is observed in cognitive process that researchers can categorize as a basic unit of thought.

Neural oscillation is an observed repetitive pattern of these energy spikes that results from the synchronization of neurons firing simultaneously. Changes in the pattern of this synchronization have been linked to perceptual and motor processes, so the underlying process that drives neurons to fire in sync merits further research as an electro-chemical mechanism for cognition. Whether it is the spike itself or the interspike interval that is most significant to the transmission of information between networks of neurons, the overall process of synchronization still has a fundamental bearing on the conscious thought process. The relatively nascent status of neurological stochastic signaling research makes experimentation on neuron spike timing difficult since statistical approaches such as principal component analysis tend not to offer insight into the actual coding strategies of neurons. This suggests that while there may be determinable correlations between neuron spikes and cognition-influenced conscious behavior, they would not be fine-grained enough to tie specific spike patterns to thought processes. Significant progress is needed in the theory of stochastic information processing before any significant conclusions can be reached.

## Cognitive Geometry: Towards a New Approach to Brain Disorders

**Fig. 1:**
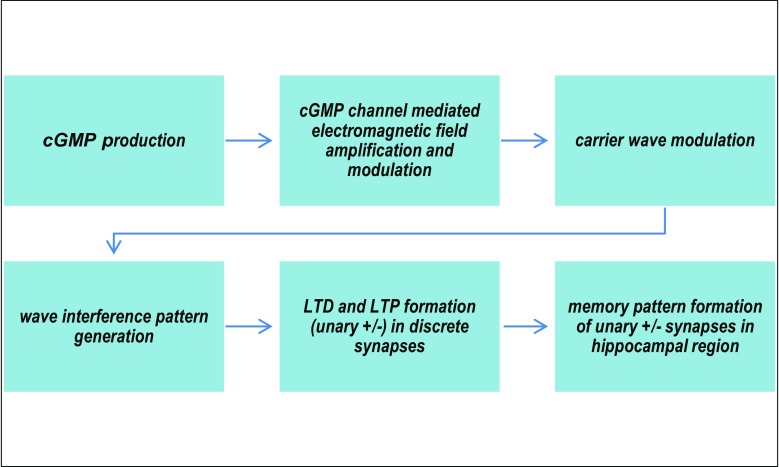
A process-level view of the Fundamental Code Unit encoding within the brain

Current treatments for cognitive disorders fall into two major categories: symptom management and blanket chemical treatment. In the former case, enough is not known about the disorder to stem the root cause, so secondary treatments aimed at alleviating the resulting symptoms form the bulk of treatment efforts. Blanket chemical treatment, on the other hand, involves the imprecise use of pharmaceuticals against a single diagnostic profile, but often with a host of side effects that result from the high usage of medication to achieve effectiveness against the original disorder. Both reflect a need for a deeper, more fine-grained understanding of cognitive disorders (Fig. 1).

To that end, we model the problem of cognitive dysfunction as one of protein structure pathology, or misfolding. Using computational cognitive research including the Unitary System and the accompanying Fundamental Code Unit (FCU), we are proposing both a new means for understanding disorders such as bipolar disorder (BD) and Alzheimer’s disease (AD), each of which are caused by the mechanisms of neurological oscillation and protein deformities, respectively. Since a protein’s structure typically provides the basis for its function, the conformational rearrangements of proteins in response to ligand binding, mutation, and covalent modification very often underlie biologically important molecular events, whether in the normal course of transducing a signal or through deleterious misfolding. The specific functions of proteins are dictated by their shapes. Thus, biologically important molecular events often consist of a change in shape or configuration of key proteins, whether as a response to ligand binding, mutation, or covalent modification. Since each of these processes introduces the possibility of deleterious misfolding, they each merit a closer examination in terms of the scope of their potential effects. Such examination will allow us to derive the cause of diseases from a closely defined set of empirical parameters instead of searching for a fit between known symptoms and protein misfolding phenomena.

Using misfolding as a causal basis, we begin to predict and explain specific changes in protein structure ranging from allosteric motion to the onset of aggregation disease. Ultimately, the primary research question is what causes the misfolding, but in our case it is treated as *miscoding*, or a deleterious alteration in the cognitive or neurobiological effects of misfolding instead of simply the physical phenomenon of misfolding. For our purposes, the question is which foldings and misfoldings serve as the correct or defective signal in the chain of Fundamental Code Unit (FCU) expressions. Then, by controlling the extant signaling mechanism through a “write” method similar to the computational equivalent, we should see changes on the macro, or folding, level. Even in the absence of abnormalities or disorders, cognition is characterized by a diversity of signals and patterns. Measurement of the ventrolateral prefrontal cortex, for instance, has demonstrated a number of significant differences in cognitive tuning functions in tasks requiring different sequencing and cognitive control demands [20].

### Case Study

As a case study, the vestibular system is a promising perspective from which to assess brain function relative to protein folding and misfolding, since it is located primarily in the mesencephalon and receives input from proprioception receptors from throughout the body. In addition, it is integrated with input from the cerebellum, semicircular canals, and visual and auditory system and relays information and coordinates the motor system to maintain balance. This serves to keep the body in a neutral position so that it is sensitive to critical environmental perturbations.

### FCU and the Uncertain Structure of Cognition

Cognition is an inherently complex, and thus necessarily uncertain, phenomenon. Specifically, the mere occurrence of a thought or brain region activation can influence future neural manifestations of the same concept. Thus, a statistically founded model is necessary, and the Maximum Entropy model is the most intuitive form [21]. For instance, Tenti et al. (2008) present a multilevel review of using computers and mathematics to model phenomena internal to the brain. Examining microscopic and molecular phenomena, along with macroscopic phenomena such as linguistics and behavioral cues, the authors attempt to elucidate the distinctions between predictive and explanatory models, and to present the problems that occur when they are used in conjunction. This paper identifies a series of problems that the FCU itself promises to address. For instance, Tenti et al. (2008) argue that, because neuro-mathematics (or “mathematical medicine,” as they call it) is so interdisciplinary and requires such collaboration, differences in expertise and background among researchers presents a number of collaboration problems. For instance, there is no common language (yet) to discern exactly what a neuro-mathematical model is, in terms of its capabilities and intended applications.

One of the most intuitive applications of Maxent to FCU is the concept set [22]. As illustrated later, apart from its intuitive network configuration that resembles the structure of cognition itself, the computational power of the FCU lies in its ability to create a “database” of concept, physical process, and linguistic linkages that tend to occur at given mind states. Populating such a database to the point that it resembles the human brain in both structural configuration and performance ability requires a method of creating connections between otherwise disparate linguistic and cognitive constructs. Berger et al. [23] and Rathnaparkhi [24] both provide some framework for using Maxent to discern (and create) relationships between concepts. Rosenfeld (1996) presents a Maxent-based concept for modeling language statistically that can be further applied to the mapping of brain region activation by S+/R− events to observable linguistic events. The basis of the author’s claim is that linear interpolation and similar approaches to adaptive language processing are deficient because they do not account for the constraints imposed by each new piece of information. The authors’ objective is to use such constraints in order to discern tendencies and patterns in natural language.

One important metric explored here is *distance*, which the authors split into short-term, intermediate, and long-term history for each new concept depending on where and when it is encountered. Distance helps to determine which conceptual components to cluster as information regarding tendencies is being gathered. Based on patterns for each of these distance requirements, the author proposes a concept known as the “trigger pair,” or a pair of word sequences whose probability of occurring within long-term history parameters is above some preset threshold. When the first phrase occurs, the second is triggered in predictive analysis, causing the probability function to change. Given a high enough probability *p*, each element in a trigger pair appears as follows:

1 $$ W:\left\{W=w\kern0.5em i.e.W\kern0.5em \mathrm{is}\kern0.5em \mathrm{the}\kern0.5em \mathrm{next}\kern0.5em \mathrm{word}.\right\} $$

2 $$ {W}_o:\left\{W\in h,i.e.W\kern0.5em \mathrm{occured}\kern0.5em \mathrm{anywhere}\kern0.5em \mathrm{in}\kern0.5em \mathrm{the}\kern0.5em \mathrm{document}\kern0.5em \mathrm{history}\right\} $$

Mutual information is then constructed between trigger pairs using a probability function, where *A* and *B* are [25]:

3 $$ I\left({A}_o:B\right)=P\left({A}_o,B\right)\frac{P\left(B/{A}_o\right)}{P(B)}+P\left({A}_o,\overline{B}\right)\frac{P\left(\overline{B}/{A}_o\right)}{P\left(\overline{B}\right)}+P\left(\overline{A_o},B\right)\frac{P\left(B/\overline{A_o}\right)}{P(B)}+P\left(\overline{A_o},\overline{B}\right)\frac{P\left(\overline{B}/\overline{A_o}\right)}{P\left(\overline{B}\right)} $$

The FCU uses a concept similar to the trigger pair in relating S+/R− events in neuronal interaction to the prime frequency oscillations that drive linguistic cognitive output. First, for each brain region identified in empirical studies, we also define a set of concepts, brain regions, and mappings between related concepts. As Howard (2012) stated previously, we begin with a set *S* (infinite) representing brain regions that may be activated by some means. From here, we take a number of additional steps to construct the concept set framework (with descriptions of symbols outlined in Table 1):

1. Introduce an *σ*-algebra. Next, we introduce a second set *W* whose elements are labeled concepts in the brain that correspond to words. For some subset A ⊂ A there is a mapping P: a ∈ A → w ∈ W called the *concept activation mapping.* The elements *a* of *A* are *action potentials.*
2. Let P̃:w ∈ W → ã ∈ Ã be a mapping we call the *brain activation mapping.* Let *μ* be a measure on *S* and let *F:A → {+,−}* be a *parity mapping.* An axiology is a mapping Ξ:W → {+,−} generated by computing *f*(*w*)=_*a*_*F*(*s*)^*d*(*μ*)^ with *a* = *P*(*w*) and then projecting Ξ(*w*) = sig(*n*(*f*)).

**Table 1:** Descriptions of symbols used in the concept set framework

Symbol	Description
*S*	Brain regions
*A*	Activation sets
*A*	Concept activation sets
*W*	Concepts
*P*	Concept activation mapping
Ξ	Axiology
*F*	Parity mapping
*μ*	Weight mapping

First we must define the composition of *S. S* is a set of sets of neurons classified according to neural topology as well as their tendency to activate in unison. Let *λ* be another weight mapping on some *S* ∈ *S*, such that *λ* assigns a greater probability of brain region activation of *S* to a subset of the neurons in *S* of size *n.* A Maxent mapping of *S* to *W* forms the initial layer of analysis, so that when *W* is later mapped to *A,* there is already a framework in existence for analyzing the brain regions and neuronal networks that become active when some concept *w* is introduced, either independently or in spoken dialog. One of the primary uses is to address statistical anomalies, such as mis-speech, momentary confusion, or other events that may skew FCU analysis in an erroneous direction. In addition, Maxent can be used to reinforce recurring set and concept activations, such as in the recall of sensory phenomena (i.e., the taste or smell of a specific food) [22, 26].

We know that neurons do not statically integrate information in the way that transistors do. In addition, the electric fields generated by neuronal activity in turn can affect that very activity. Thus, since the binary mathematical principles that guide discrete transistor-based computation do not map particularly well to the brain, and it is necessary to seek a more holistic view, hence the FCU. The Fundamental Code Unit (FCU) seeks to unify multiple sensor data streams, such as linguistic input which is considered here, neurological data (i.e., cell and network activation, and neural firing rate and amplitude), and behavioral phenomena (i.e., nervous tics, spatial judgment errors, and gait irregularities) into a single computationally efficient framework [27]. Because data collection methods will never achieve perfection, and due to the inherent uncertainty in physiologically complex processes such as cognition, there will always be some degree of missing or conflicting information in FCU classification and construction. To that end, maximum-entropy statistical models can be used to account for this uncertainty, as well as provide innovative Big Data predictive analytical-based capabilities (Hussain and Newton et al. 2014 [28]) for events that have yet to take place.

## Implications of a Unit-Based Approach

### Axiology of Human Thought: The Unitary System

The brain behaves in many different ways; its outputs manifested in physical movements and behaviors. Language, an expression of behavior, is thus a useful tool in analyzing brain function. We posit that there is a transition from the molecular to the behavioral expressions of interactions of brain functions such as the stochastic signaling phenomenon discussed in the previous section, which translates to language, and that a better understanding of axiology can disambiguate this process transition. The fundamental distinction within all languages that our work focuses on (i.e., between semantic primes and non-primes or idiomatic words/expressions) is significant in that the cognitive processes leading to their acquisition are also distinct; thus, conceptual divides such as these, both in linguistics and psychology, provide important patterns to search out in the process of tying this concept of semantic primitives to the notion of stochastic signaling.

Axiology is a direct, multi-faceted description of a structure and function of the human language. It can aid us in understanding perceptions, as well as in unifying the biochemical and cognitive structural aspects of our understanding of conscious thought. The axiological model creates a code, the *G* code, which is unified and omnipresent within brain function. The *G* code defines the “how” of each action in the outcome of the brain’s decisions, as the code is being generated and executed on different mediums, but produces the same outcome: language. Thus, in order to dissect this code, it is useful to analyze language, which is based in axiology.

The central problem of axiology consists of issues in value and value theory. Values make up polar pairs; they are clear-cut dichotomies. They are either positive or negative, and the determination is made by intuition and inference. That which is intrinsically valuable is that which is inherently good, while that which is extrinsically valuable has value instrumental to something else. We extend this problem to include the creation of a mapping of axiological values to neurological state changes; in this way, two interdependent levels of cognitive analysis will be unified and provide a clearer picture of the components and structure of cognition.

**Fig. 2:**
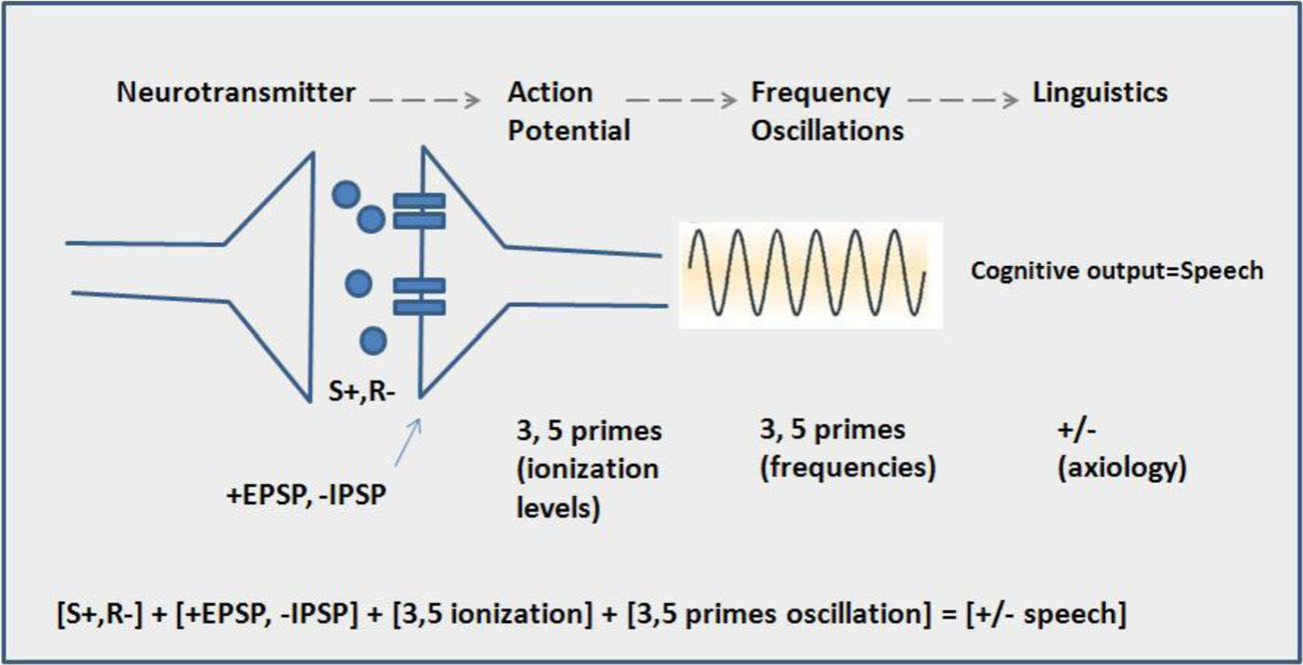
Multilevel code translation from neuronal interaction to cognition and language

Axiological semantics involves an examination of values with reference to the meaning of various linguistic expressions. The task of axiological semantics is to describe values and the manner in which they determine the structure and functioning of the human language as manifesting in communication. Krzeszowski [29] contended that preconceptual image schemata (which provide a format for encoding information from vision and language simultaneously) include the axiological parameter PLUS-MINUS or positive-negative (i.e., all iage schemas, like values, exhibit a bipolar property of conferring positive or negative associations). All schemas can be understood to have euphoric or dysphonic characteristics. Axiological concepts emerge from axiological poles of preconceptual schemas through metaphorical extensions. Consequently, all words and linguistic symbolic units are assessable on an axiological scale. Even if words seem to be axiological neutral, they are still prone to axiological distinction given appropriate contexts (Fig. 2).

There is a formal scientific way to identify and rank human values, achieving values appreciation, values clarification, and values measurement since they are cognizable. The experience of value involves feeling, volition, desire, and an acquaintance with the object of value; this is a noetic feature. If considered coordinates of human actions—and when their finality is distinguished from their efficiency—qualitative values may become, within some limits, quantifiable. However, the primary relational determinant of value is polarity. Thus, we must define an axiological parameter [1].

Some scholars assume that the concept of a value is explicable only in terms of good, bad, and indifferent. They surmise that an individual with a “positive” state of mind is happy and content, while an individual with a “negative” state of mind is sad and unsatisfied. This interpretation undermines the idea that the use of language extends beyond its semantic features and functions and overlooks the principle that we have various apparatuses for construal. Therefore, the positive-negative evaluation cannot be limited to good-bad and the happy-sad scale, for it is only one dimension of the axiological parameter. Thus, for the purpose of our study, we also consider the time orientation schema. We have proposed “positive” can be analogous to “future-oriented” and “negative” can be analogous to “past-oriented.”

As human beings, we are uniquely motivated to orient ourselves in relation to time. It is our sense of future-ness that enables us to mature beyond a psychopathic stage [30]. Several cognitive psychologists focus on the parallel nature of memory and future thinking as processes involving reliving the past and pre-living the future. For example, Lewin maintained that an individual’s actions, emotions, and morale depend on his or her aspirations in relation to time perspective [31]. Lewin underscored the motivational power of constructed future images and their development across age [32]. Similarly, Zimbardo suggested than an individual’s attitude towards time might be as significant as personality traits like optimism or sociability when assessing mental state [33]. Time perspective influences our judgments, decisions, and actions. Since linguistic expression corresponds to mental state, we adopt the logical assumption that words reflect our time perspective (Seginer, 2009).

To develop a “default axiology,” we first determine the “intrinsic value” of words that have been characterized as semantic primes, based on the positive-negative axiological parameter. A semantic prime is a linguistic expression whose meaning cannot be presented in any simpler terms. It has a lexical equivalent in all languages. In cases where the intrinsic value of a word is ambiguous, it is useful to consider the antonym of the word to determine its value. To determine the axiological value of linking and auxiliary verbs, we considered the “temporal value” of words based on the past-future scale. While intrinsic value of these verbs may be ambiguous, we can still assign a positive or negative grammatical axiological value to these words. Verbs in the future tense indicate future-oriented perspective and should be designed as positive. Verbs in the past tense indicate past-oriented perspective and should be noted as negative.

We can also determine the “consequent value” of words for which the intrinsic value is not immediately apparent. For example, closed class words, or those that are less definable in terms of other words, have less readily identifiable meaning and thus, it is difficult to assign an axiological value to these words. However, the value of surrounding words in the passage provides context that can be used to determine value. Consequently, the cognitive model suggests that a grammatical subsystem can also be semantically characterized. This view entails a continuum between open- and closed-class within the knowledge base. Thus, the value of closed-class words can be determined by their context within a phrase or sentence.

However, when people speak, they combine words into sentences. Thus, it becomes necessary to determine the positive or negative value of an expression and derive aggregate values. The linguistic constructs that describe values present as manifestations of the brain structures that are involved in generating and analyzing discourse and ultimately behavior. Linguistic models, such as LXIO, a mood analyzer system for discourse, enable us to determine the values of expression in language, thus providing insight into brain structure and function.

### Molecular Chirality and Cognition

**Fig. 3:**
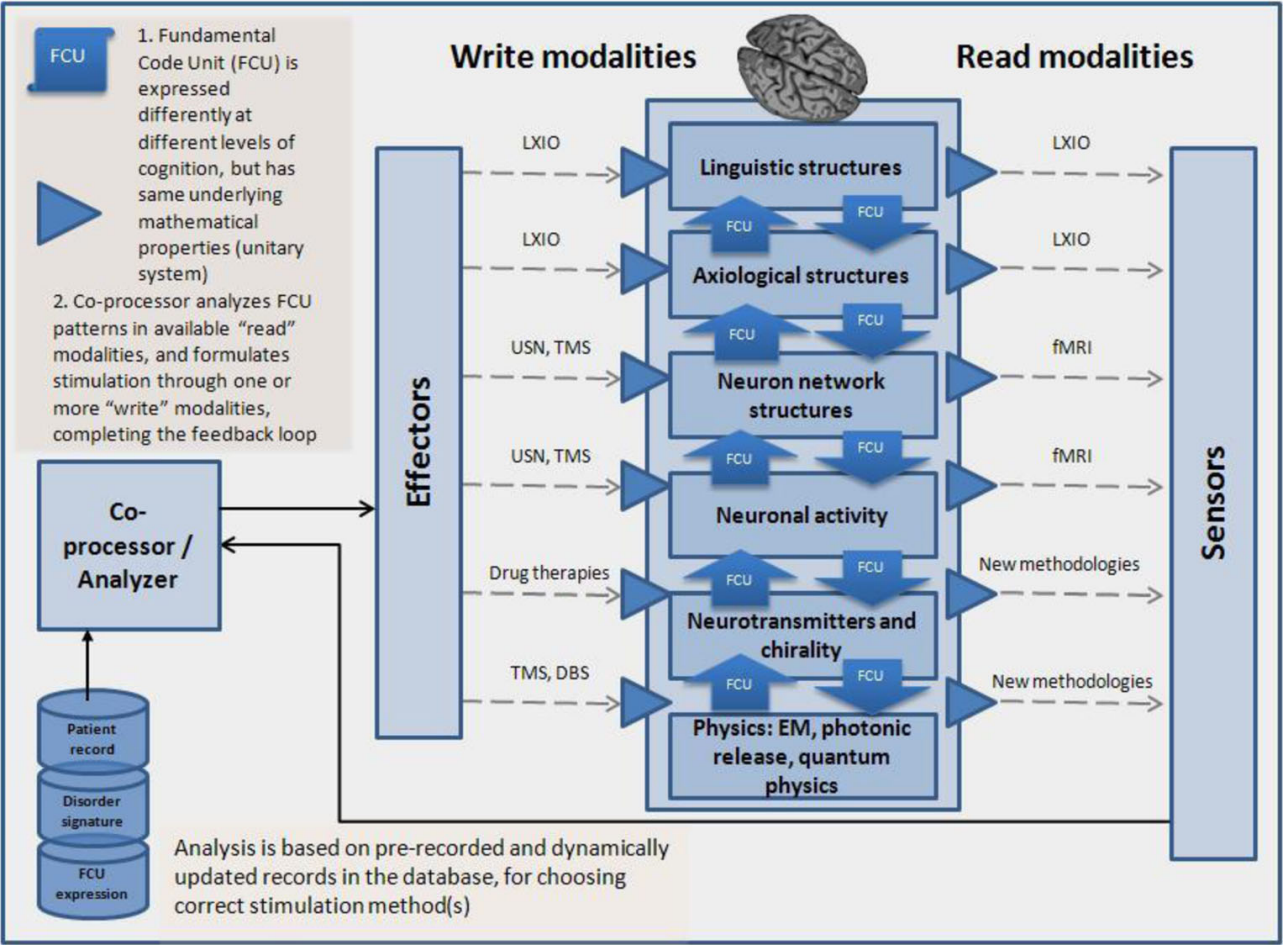
Multi-level neurofeedback using FCU analysis

One can see the unitary system functioning at the molecular level in the molecular chirality concept. At a synapse, a neuron releases neurotransmitters that excite or inhibit another cell or alter its response to other input. Excitatory neurotransmitters, the most common type, increase the firing rate. An inhibitory neurotransmitter decreases the chances of the neuron firing. This is the most common type, while still others increase firing rate (or the chances that the next neuron will fire). Each neuron is influenced via multiple neurotransmitters acting at multiple synapses by dozens of other neurons. If we assume that neurotransmitters are composed of the 22 proteins, are used in biology (and the number may be smaller), and let L be the average number of proteins in a neurotransmitter, then the entropy of the neurotransmitter space is 22L. The relationship of neurotransmitter geometries to one another and to geometry of neuroreceptors gives rise to complex absorption behavior. A tight match can lead to reliable high rates of uptake, while less perfect matches can lead to lower and/or more stochastic uptake behavior. The use of particular sequences of wave of transmitters can condition rates of uptake (Fig. 3).

Following the release of a neurotransmitter and the subsequent activation of a receptor, it is important that the response is terminated and the system reset so that a subsequent activation can occur. This is achieved via the removal of the neurotransmitter by metabolic enzyme activity and by passive or active uptake activities [34]. The concentration of the transmitter at the synapse for a longer time period occurs if the uptake mechanism is blocked. Therefore, a neurotransmitter uptake blocker may have an effect similar to a postsynaptic agonist of that transmitter. For uptake to take place, the neurotransmitter must be recognized by an uptake mechanism. As a result, it is common for structural analogs of the neurotransmitter of this process, for example, noradrenaline, serotonin, and dopamine. Once again, we see the unitary system at work in the form of chirality, as explained next.

Biological molecules such as neurotransmitters exist in mirror image isomers of one another, and this is what governs the ligand-substrate interaction specificity necessary for biochemical reactions [35, 36]. The recognition of a neurotransmitter by its complementary receptor occurs due to the unique conformation of each isomer-enantiomer ligand, functioning in a lock and key mechanism. The two mirror image isomer-enantiomer molecules will interact with postsynaptic receptors, producing different biochemical effects. The ligand-substrate specificity required for neurotransmitter activity is conferred by the unique electronic interactions between asymmetric molecules [35]. The all-or-nothing action potential is generated by the summation of excitatory and inhibitory signals in the form of chiral molecules that are found in a *S* (+) or *R* (−) isomer-enantiomer conformation binding and releasing from the receptor, generating a strong enough signal to cause the neuron to reach threshold. While the chiral neurotransmitters are mirror images of one another, it is important to remember that they are not the same molecule and will not exert the same strength of signal upon the receptor. The salience of the signal is determined by the strength of the ligand-substrate interaction [37].

While it may not be the case with regards to all stimulant actions, particularly those produced in the periphery, such as cardiovascular actions, with respect to the central stimulant actions on a regional structure of the central nervous system, the *S* (+) isomer is several times more potent than its *R* (−) enantiomer. The *S* (+) isomer is known to induce euphoria, whereas enantiomer *R* (−) causes depression. The overall greater potency of the *S* (+) isomer form with respect to central stimulant actions suggests that this form may have a higher potential for deep cranial stimulant actions and neurotransmitters availability in the synapse. This leads to behavioral- alteration notice on corresponding linguistics states. The correlations of the LXIO system and *S* (+) isomer and *R* (−) enantiomer values offer corresponding equivalence of transporter’s chemical pathways. Thus, mood states are viewed at the molecular and behavioral level, whereas prior research only proposed framework for neuronal correlation based on constellations activity in imagery. Addition of *R* (−) enantiomer into pharmaceutical treatment provides them with a quicker onset and longer clinical effect compared to pharmaceuticals exclusively formulated using the *S* (+) isomer. Nevertheless, it seems the human brain favors *S* (+) isomer over *R* (−) enantiomer. Central stimulants exert their effects by binding to the monoamine transporters and increasing extracellular levels of the biogenic amines dopamine, norepinephrine, and serotonin.

The *S* (+) isomer acts primarily on the dopaminergic (DA) systems, while *R* (−) enantiomer acts primarily on norepinephrinergic (NE) systems. Consequently, the primary reinforcing and behavioral-stimulant effects of the *S* (+) isomer are linked to enhanced dopaminergic activity, primarily in the mesolimbic dopaminergic pathway. *S* (+) isomers bind to the dopamine transporter (DAT) and blocks the transporter’s ability to clear DA from the synaptic space. In addition, the *S* (+) isomer is transported into the cell, which leads to dopamine efflux (DA is transported out of the cell and into the synaptic space via reverse transport of the DAT). In high doses, the *S* (+) isomer and *R* (−) enantiomer can also inhibit the enzymes monoamine oxidase *A* and *B* (MAO-A and MAO-B). MAO-A is responsible for breaking down serotonin, dopamine, norepinephrine, and epinephrine. MAO-B is responsible for breaking down dopamine (more potently than MAO-A) and phenylethylamine (PEA), which has actions similar to the *S* (+) isomer itself, and is thought to be involved in feelings of lust, confidence, obsession, and sexuality. The ability of *S* (+) isomer, and to a lesser extent *R* (−) enantiomer, to inhibit MAO-A and MAO-B, results in the accumulation of monoamines. Thus, central stimulant actions directly excite the release of these neurochemicals, which results in a potent elevation in monoamine neurotransmission. In sum, the effect of a central stimulant, the *S* (+) isomer and to lesser extent *R* (−) enantiomer, is to increase neurotransmitter availability in the synapse, by both releasing more neurotransmitters, as well as prolonging their availability in the synapse by slowing their removal.

The *S* (+) isomer and *R* (−) enantiomers are the most important part of the chemical exchange at the molecular level of neurobiological information exchanges. By studying them, we can explain the structure that is present in the fundamental code unit, as well as better understand which parts of stochastic signaling (i.e., energy spikes or spike intervals) most closely correlate with conscious cognitive expression. Ultimately, these molecules that govern molecular informational exchanges are the physical mechanisms by which the Unitary System manifests itself in the human brain; using unary mathematics we create a model by which the chemical and physical aspects of cognition are converted into a coding of human thought that is both computationally meaningful and understandable to humans.

## Genetic Vs. Cognitive Information Storage: Links and Applications

Human cognition is an inherently physical process. Given enough time and sufficiently sensitive instruments, human thoughts could be reduced to patterns of electrochemical as well as atomic and subatomic phenomena. However, as the recent Kurzweil debate has shown, cognition is much more complex than the sum of its parts. To rely upon instrumentation alone in the quantification of cognitive phenomena would be to misunderstand the sheer complexity of the brain’s internal structure and function. The brain’s structure is derived from genetics, but genetics do encode the layers of complexity added by experiences, sensations, and new connections. These neural networks are densely interconnected which are thought to play vital roles in cognitive functions and information integration [38]. To that end, our research is a codification of the most basic unit of cogent thought, from which conscious thoughts can be assembled.

## Summary

The Fundamental Code Unit of Thought (FCU) is an attempt to bridge this gap between the physical phenomena we observe and their complex results. The FCU consists of two essential components. The framework itself can also be considered a trans-disciplinary informational container that spans a number of analytical and physical dimensions. Due to the fact that cognition is both a physical and computational (i.e., conceptual) occurrence, any benchmark by which we hope to measure it must take both of these into account. As Howard (2012) argues [39]:

The underlying units that compose cognition, like those of DNA, are relatively simple compared with the structures they create. This applies both to the brain itself and the way we perceive it (i.e., as a system of sensory inputs and linguistic and behavioral outputs). In our approach, we map the physical phenomena of cognition to this theoretical system.

In addition to the FCU, our conception of brain language includes the mathematical framework in which the FCU is located. The Unitary System is founded on unary mathematics. The functions “unary plus” (+) and “unary minus” (−), representing an increase or decrease in the underlying measured value, carry sufficient computational efficiency to represent human cognition, provided that the same linguistic base is present on both sides. The brain communicates within itself and with the rest of the body via unitary operators. These unitary operators carry a state of time and space that conveys information necessary to decipher any semantic or non-semantic based language. Because these operators are language-agnostic, they provide a common language of cognition when the FCU is applied.

DNA and its cognitive equivalent, the Unitary System/FCU, are not only conceptually linked but physically linked as well. In a recent paper published by the Proceedings of the National Academy of Sciences, this concept is examined in more detail, in a “rewriteable recombinase addressable data (RAD) module [40] that reliably stores digital information within a chromosome.” How is genetic information storage tied to cognitive information storage? Each has a distinct biological foundation (the latter is driven by recombinase proteins), so the dimensionality of the information is similar, as is the stochasticity: “stochasticity in RAD system performance arising from bidirectional recombination can be achieved and tuned by varying the synthesis and degradation rates of recombinase proteins.”.

## Conclusions and Outstanding Challenges

The ability to use existing biological structures such as DNA and proteins to store information means that biological bit-encoding is increasingly feasible, despite the fact that the brain has been performing this process for eons.

This position paper has outlined how using unary mathematics, and unifying external data with internal processes, can help achieve the outcome of pertinent thoughts in opposite situations—the most complex decision making process performed by the brain. Current and future research is aimed at experimentally testing and validating the FCU, for further setting the foundation for future hypothesis found in contemporary research and the theories underlying it [41, 42]. In particular, significant underlying theoretical challenges still need to be addressed, in order to realize FCU’s ambitious aims of unifying multiple cognitive sensory data in real-time, by fusing linguistic input with relevant neurological data and behavioral phenomena, into a single, next-generation cognitive framework.
